# A novel smartphone application for early detection of habanero disease

**DOI:** 10.1038/s41598-024-52038-y

**Published:** 2024-01-16

**Authors:** Ronke Seyi Babatunde, Akinbowale Nathaniel Babatunde, Roseline Oluwaseun Ogundokun, Obiwusi Kolawole Yusuf, Peter O. Sadiku, Mohd Asif Shah

**Affiliations:** 1 Department of Computer Science, Kwara State University, Malete, Kwara State Nigeria; 2 Department of Multimedia Engineering, Kaunas University of Technology, Kaunas, Lithuania; 3 Department of Computer Science, Landmark University Omu Aran, Omu-Aran, Nigeria; 4 Department of Mathematics and Computer Science, Summit University Offa, Offa, Nigeria; 5 Department of Computer Science, University of Ilorin, Ilorin, Nigeria; 6 Department of Economics, Kebri Dehar University, Kebri Dehar, 250, Somali Ethiopia; 7 Centre of Research Impact and Outcome, Chitkara University Institute of Engineering and Technology, Chitkara University, Rajpura, 140401, Punjab India; 8 Division of Research and Development, Lovely Professional University, Phagwara, 144001, Punjab India

**Keywords:** Plant sciences, Imaging

## Abstract

Habanero plant diseases can significantly reduce crop yield and quality, making early detection and treatment crucial for farmers. In this study, we discuss the creation of a modified VGG16 (MVGG16) Deep Transfer Learning (DTL) model-based smartphone app for identifying habanero plant diseases. With the help of the smartphone application, growers can quickly diagnose the health of a habanero plant by taking a photo of one of its leaves. We trained the DTL model on a dataset of labelled images of healthy and infected habanero plants and evaluated its performance on a separate test dataset. The MVGG16 DTL algorithm had an accuracy, precision, f1-score, recall and AUC of 98.79%, 97.93%, 98.44%, 98.95 and 98.63%, respectively, on the testing dataset. The MVGG16 DTL model was then integrated into a smartphone app that enables users to upload photographs, get diagnosed, and explore a history of earlier diagnoses. We tested the software on a collection of photos of habanero plant leaves and discovered that it was highly accurate at spotting infected plants. The smartphone software can boost early identification and treatment of habanero plant diseases, resulting in higher crop output and higher-quality harvests.

## Introduction

Habanero peppers are an essential crop grown in many regions worldwide, known for their unique flavour and spiciness^1^. However, like all crops, habanero peppers are susceptible to various diseases, leading to significant yield losses if not detected and treated promptly^2^. Over 80% of people's food comes from plants, which are also cattle's primary nutrition source. Pests and plant diseases frequently threaten the availability and security of plants for human and animal consumption. Up to 30% of the world's primary staple crop yields can be lost, costing thousands of dollars in lost food supply^3^. Around 16% of the global crop yield loss is attributable to plant diseases^4^. Visual inspection of the plants has historically been used to identify conditions. Still, this method can be labour- and time-intensive and is also influenced by the observer's level of competence^5^. With the rise of deep learning techniques and smartphone technology^6^, there is an opportunity to automate and streamline the process of plant disease detection^7^, disease detection^8−10^ and so on, improving the accuracy and speed of disease diagnosis and management.

Early blight is a fungal disease that explicitly affects tomatoes but may also impact potatoes, peppers, and eggplant. Plants are susceptible to early blight at any point during the growth season. It is pervasive in areas with high humidity and temperatures over 75°F. The infection is disseminated by the air or insects, which transport fungal spores. Spores undergo germination and develop when plants are saturated with moisture due to copious dew or frequent precipitation. The first signs of early blight manifest mainly on the more aged and fully expanded leaves close to the plant's lower region. Each leaf has one or two uneven dots. The dots have a diameter ranging from ¼ to ½ inch. The dots are target-like, characterized by tan cores with concentrically ridged rings and yellow halos around the margins. The boundaries of the spots are well-defined. The sites increase in size and merge. A significant leaf area undergoes widespread yellowing and may subsequently detach.

The disease is seen in the United States and Canada, with more severe cases occurring in humid locations, particularly in the Midwest and eastern states. Early blight manifests as depressed lesions on the fruit's stem in tomatoes, eggplants, and peppers. Spots have a concentric ring pattern or a target-shaped appearance. The pathogen spreads from the branch to cause decay in the fruit.

In this study, we describe the creation of a smartphone app based on a Convolutional Neural Network (CNN) model for identifying habanero plant diseases. The created method enables farmers to take pictures of infected habanero plants using their smartphones and then utilize the CNN model to determine whether the photographs are healthy or infected and, if infected, to determine which disease is present. The smartphone application may connect farmers with agricultural specialists for more advice and provide information on suggested treatments for each infected plant diagnosed.

Recent years have seen a rise in interest in applying deep learning models to detect plant diseases^11−14^. Numerous research has shown these models' ability to increase the precision and effectiveness of disease detection^15−18^. Only a few studies have been published^19−21^, making developing smartphone apps^22^ specifically for plant disease detection a relatively young field. Our study expands on these earlier studies by creating a smartphone app specifically for diagnosing habanero plant diseases and analyzing its performance on a collection of photos of habanero plants.

The remainder of the document is structured as follows: Section “Related Works” provides an overview of a review of related work on developing smartphone apps for plant disease detection. Section “Materials and Methods” describes the dataset used to train and test the CNN model. Section “Materials and Methods” presents the methodology for developing the smartphone app and training the CNN model. Section “Results and Discussion” provides the results of our tests on both the mobile app and the CNN model. Section “Limitations of the Study” discussed the study's limitations and how it can be improved. Implications of our research and directions for future work are discussed in the last section of the paper, Section “Conclusion”.

## Related works

Deep learning algorithms have been used in various fields for detection, such as^23^, surgical instruments^24^, outlier detection^25^, constructing agricultural information systems^26^ and so on. The most prevalent architecture for plant disease diagnosis using deep learning (DL) algorithms is CNN, which has shown encouraging results. Applications that rely significantly on image analysis, such as plant disease diagnostic, may benefit from CNNs because of their ability to automatically learn properties from images and classify them into a wide variety of categories. CNNs have been investigated for their potential in diagnosing plant diseases in recent research^11−14^, with encouraging results.

Smartphones have also emerged as a powerful tool for simplifying and increasing access to plant disease detection. Apps that utilize a smartphone's camera to diagnose infected plants by analyzing phones using deep-learning models are already widely available^19, 21^. These applications have the potential to significantly enhance the pace at which infected plants may be diagnosed while also reducing the cost and level of expertise required for diagnosis.

Several smartphone applications for plant disease detection have been developed recently, such as Plantix, Nuru, and Crop Doctor^19−21^. These apps use a variety of deep learning models, including CNNs, to classify images of infected plants and provide treatment recommendations. To create an automated diagnosis and identification method for leaf spot-infected plants in all three sugar beet ailment severity categories (mild, moderate, and severe), Ozguven and Adem^27^ revised the Faster R-CNN by boosting the depth of the data layer from 32 × 32 to 600 × 600 pixels. The created Faster R-CNN outperformed the Faster R-CNN with an accuracy of 95.48 per cent. A method of categorization for the degree of severity of crop infections and bug pests was established by Yu et al.^28^, who set out to address the issue that the algorithm used for classification was not satisfactory by proposing a better ResNet50 framework (CDCNNv2) that utilized DTL. The technology does more than identify crop pests and infected plants in real time; it also offers advice on how to avoid and treat them and what medications to use.

The PARNet framework and accompanying WEB interface were developed by Li et al.^29^, who fused the attention technique with the residual architecture to achieve a significant effect. The method has a typical accuracy of 96.84% in detecting five infected plants affecting tomato leaves. It outperformed the VGG16, ResNet50, and SENet algorithms by 2.25%-11.58%. Jiang et al.^30^ suggested a CNN framework for ginger-infected plant detection using four types of ginger ailment gathered in their natural habitats, and they did so by redesigning and optimizing the CNN architecture centred on the conventional LeNet-5 network. The diagnosis accuracy for four distinct infected plants affecting ginger was 96%.

Using DTL and the Faster R-CNN, Zhou^31^ identified five types of apple leaf infected and created an Android-based device to detect them. For apple leaf infected, the algorithm saw them with a typical identification accuracy of 76.55%. When Liu et al.^32^ implemented the MobileNet model on a mobile phone, they achieved a typical identification accuracy of 87.5% for six types of infected grape leaves gathered in the real world, with a mean computation time of 134 ms per imagery.

Esgario et al.^33^ built a device using the ResNet50 framework to detect and quantify the impact of environmental stressors on coffee leaves. The method’s categorization of the effects of biological stressors on coffee leaves had an accuracy of 95.24 per cent, and the degree of stress estimate accuracy was 86.51 per cent. Xiong et al.^34^ suggested an automated picture segmentation technique founded on the GrabCut methodology to create a crop-infected identification tool for mobile intelligent gadgets^35^. They chose the MobileNet as the DL categorization approach. Over 80 per cent of 27 infections across six crops were correctly identified by the technology in the testing environment and the real world.

Bezabih et al.^36^ suggest employing a merged neural network that combines the retrieved characteristics from VGG16 and AlexNet networks. This approach aims to create a better infection classification model using fully connected layers. The proposed concatenated CNN model involves many processes, including dataset collecting, picture preprocessing, noise reduction, segmentation, feature extraction, and classification. The suggested concatenated CNN model was assessed, yielding a training classification accuracy of 100%, validation accuracy of 97.29%, and testing accuracy of 95.82%. Overall, the study's results suggest that the suggested concatenation model using digital photos is well-suited for diagnosing infection in pepper leaves and fruits.

Kumar, Razi, Singh & Das^37^ have introduced a novel model named Res-VGG, which combines two distinct deep-learning models, VGG16 and ResNet. This approach has been used to identify and classify the symptoms of plant diseases. Our suggested model has 12 layers comprising nine convolutional layers, two fully connected layers, and one softmax layer. The efficacy of this proposed model has been assessed and confirmed using the Plant Village dataset.

Pant et al.^38^ presented a study paper suggesting that convolutional neural networks (CNN) provide a more effective solution for controlling disease in capsicum leaves. The proposed CNN model demonstrates good accuracy in validation and a quicker convergence rate.

Researchers have proposed using Convolutional Neural Networks (CNN) to address these challenges for categorizing and detecting plant diseases based on images. The authors have specifically studied the capsicum plant, sometimes known as the Bell pepper, which is a member of the Grossum cultivar group within the species Capsicum annuum.

Once the model has been developed and fitted, the operational performance and quality may be assessed using a testing dataset that has not been previously observed. The performance is quantified based on its level of precision. The model accuracy of each block in the VGG model may be determined by augmenting the number of convolutional layers and pooling layers. The model's accuracy has been enhanced from 84 to 96%. This research utilizes a convolutional neural network to detect, diagnose, and categorize diseases in capsicum plants. This research report explored three distinct enhancements to the basic model. The performance of the various outcomes may be succinctly stated in terms of model accuracy.

In this work, we want to narrow this gap by creating a smartphone app based on a CNN model for detecting habanero plant disease. We test the app and the CNN model on a dataset of habanero plant photos and compare our results to previous research in plant disease diagnosis using deep learning methods. To our knowledge, no smartphone applications are currently available for detecting habanero plant diseases.

## Materials and methods

### Dataset

We gathered a dataset of habanero plant images, including 1478 images of healthy plants and 997 photos of plants with one of five common diseases: bacterial spot, anthracnose, powdery mildew, Phytophthora blight, and tomato spotted wilt virus as obtained from the Kaggle repository with link: https://www.kaggle.com/datasets/arjuntejaswi/plant-village?resource=download. We used this dataset to train and test our CNN model. The images were collected from multiple sources, including field surveys and online plant pathology databases.

Before Analysis, the dataset was preprocessed by scaling each image to 224 × 224 pixels and standardizing the pixel values to be between 0 and 1. 60% of the photos were utilized for training, 20% for validation, and 20% for testing after the dataset had been randomly divided into these three sets.

### Proposed MVGG16 model

The study proposed a deep transfer learning model VGG 16, which was optimized by fine-tuning some layers and adding dropout and regularization techniques to prevent overfitting. The study also employed the transfer learning method because of the small dataset obtainable. In Section V, we provide the results of our tests on both the mobile app and the CNN model. Lastly, section VI uses the picture of an infected leaf to diagnose a plant. Some leaf functions, such as recognizing infection, are associated with a particular colour, and this hue may change according to the procedure. Here, we put leaf photos through some deep learning algorithms to predict the plant disease name based on the colour of the splits in the broad leaf, which signal the presence of a disease.

The CNN model utilized in this investigation was based on the VGG16 architecture, which has been extensively used in earlier studies to identify plant diseases^12−14^. Using the Keras deep learning framework, the model was put into practice. It was trained using stochastic gradient descent (SGD) on the habanero plant dataset with a learning rate of 0.001 and a momentum of 0.9. The model was trained for 50 epochs with dropout and regularization (L2) to avoid overfitting. The smartphone app was developed using the Flutter framework, which allows for the development of cross-platform apps for both Android and iOS devices. The app will enable users to capture images of habanero plants using their smartphone phones, then use the trained CNN model to classify the images as healthy or infected and, if infected, to identify the specific disease. The app also provides information on recommended treatment options for each identified condition and can connect farmers with agricultural experts for further guidance.

The VGG16 is a well-known convolutional neural network (CNN) with a total of sixteen layers, thirteen of which are convolutional layers and three fully connected layers. The design is distinguished by the fact that it employs 3 × 3 convolutional filters with a stride of 1 to extract distinguishing characteristics from the pictures fed into it. Max-pooling layers are used to decrease the size of feature maps while still preserving the necessary information. ReLU activation functions introduce non-linearity, and the final fully connected layer generally corresponds to the number of classes being classified in the classification job. Throughout the training, dropout layers randomly deactivate neurons, which helps to prevent overfitting throughout the process.

Pretraining is an essential phase in the transfer learning process, providing its context. The VGG16 model is first pre-trained on a massive dataset such as ImageNet, enabling it to learn broad visual properties and patterns without further training. After completing the pretraining phase, the model is then fine-tuned for a particular purpose, such as identifying infections in habanero plants. This method of fine-tuning entails adjusting the architecture of the model and the weights so that they align with the categories of the target dataset. It is possible to extend the size of the training dataset by using data augmentation methods such as zooming and rotating the data inside the dataset. Classification accuracy is measured by using an appropriate loss function, such as categorical cross-entropy.

Additionally, the basis of the VGG16 architecture is leveraged by the VGG16 DTL model that was applied in the study. After pretraining on a broad dataset, it is then fine-tuned for the habanero plant disease detection job by using a particular dataset and approach. The model's capacity to accurately classify habanero plant infections is improved by using transfer learning, which enables the model to benefit from information obtained from a wide variety of photos.

### Modification made on VGG16

A number of standard modifications were likely made to adapt the VGG16 architecture for detecting habanero plant diseases. In the first place, the original final output layer would have been changed with a tailored output layer to match the number of classes in the habanero plant disease dataset. This specific output layer often comprises neurons representative of each infected group. The researchers may have also modified a number of the convolutional layers of the VGG16 algorithm to improve their ability to identify characteristics of habanero plant infections. The updated data preparation would have included data augmentation strategies and input image scaling techniques specifically customized to habanero plant photographs. In order to achieve the highest possible level of performance, hyperparameters such as learning rate, batch size, and optimizer would have been fine-tuned.

Additionally, regularization techniques like dropout or L2 regularization may have been used to reduce the amount of overfitting. It would have been essential to use an appropriate loss function, such as categorical cross-entropy, to classify diseases affecting habanero plants accurately. During the training phase, several different measures could be taken. These included adjusting the number of training epochs, implementing early stopping, and monitoring the model’s performance on a validation dataset. According to the particular infection detection target, using domain-specific information or rules in creating the model or the post-processing procedures to increase accuracy is contingent upon the model.

### Ethics approval

The dataset used in the study was obtained from a public repository (Kaggle); therefore, ethical approval from the ethical committee is not applicable here.

### Informed consent

The research does not require gathering datasets or using humans, so this statement does not apply to this study.

## Results and discussion

### Preprocessing

**Figure 1:**
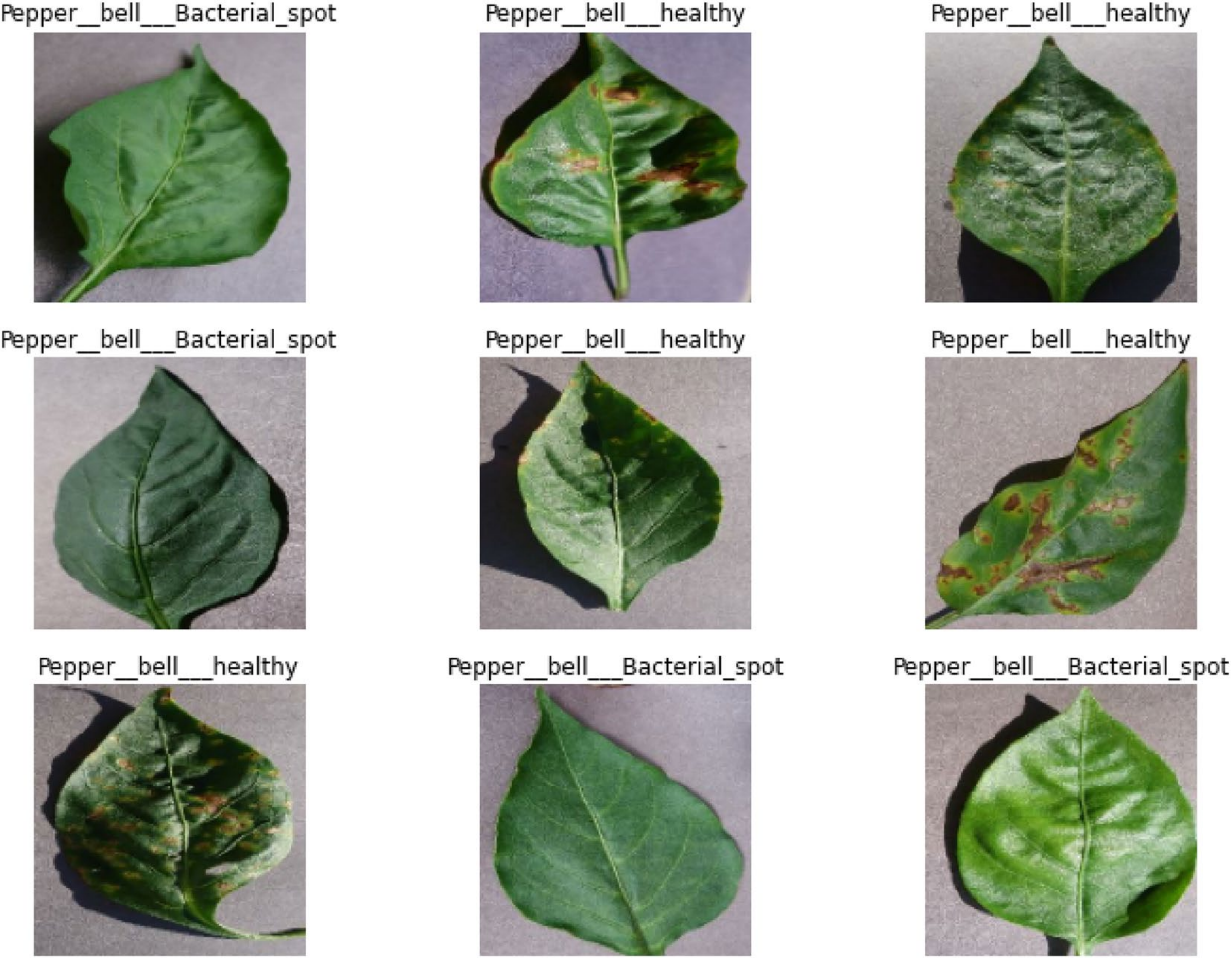
Sample of the images used for the experimentation.

We collected a dataset of habanero plant images that included healthy plants and those infected with various diseases. A total of 2475 images, 1478 for healthy plants and the other 997 for infected ones, made up the dataset. We manually classified the photos for the classification test to establish ground truth labels. Four hundred and ninety-five images (20%) were used for validation, another four hundred and ninety-five images (20%) for testing, and one thousand four hundred and eighty-five images (60%) were used for the training set. The images were preprocessed in several ways, such as shrinking them to 224 × 224 pixels, making them grayscale, and levelling the pixel values. Additionally, to broaden the variety of the training data, we used data augmentation techniques, including random rotation, flipping the horizontal and vertical axis, and zooming. Figure 1 visualizes the sample of the images used to implement the proposed system.

### Simulation environment

Results were acquired from the developed system after training and execution of the development of a smartphone app for habanero plant disease detection using a laptop computer with 16 GB RAM, an Intel(R) CoreTM i5-9300H CPU running at 2.40 GHz, Google Collab, and Python 3.0.

### Performance evaluation

We employed many metrics, including accuracy, precision, recall, and F1 score^39−41^, to evaluate the performance of our model. We determined the accuracy by dividing the total number of images by the percentage of correctly classified images; the formula^42−44^ can be seen in Eq. (1). We defined precision as the ratio of true positives, or plants correctly identified as having a disease, to all predicted infected plants, as shown in Eq. (2). The percentage of genuine positives to all actually infected plants was found to equal the recall (See Eq. 3). The F1 score is created by harmonically summing precision and recall, which is represented in Eq. (4).

1 $$Acc = \frac{(TP + TN)}{{(TP + FN + FP + TN)}}$$

2 $$\Pr e. = \frac{TP}{{(TP + FP)}}$$

3 $${\text{Re}} c. = \frac{TP}{{(TP + FN)}}$$

4 $$F1 - score = \frac{(2*pre.*rec.)}{{(pre. + rec.)}}$$

## Results

**Figure 2:**
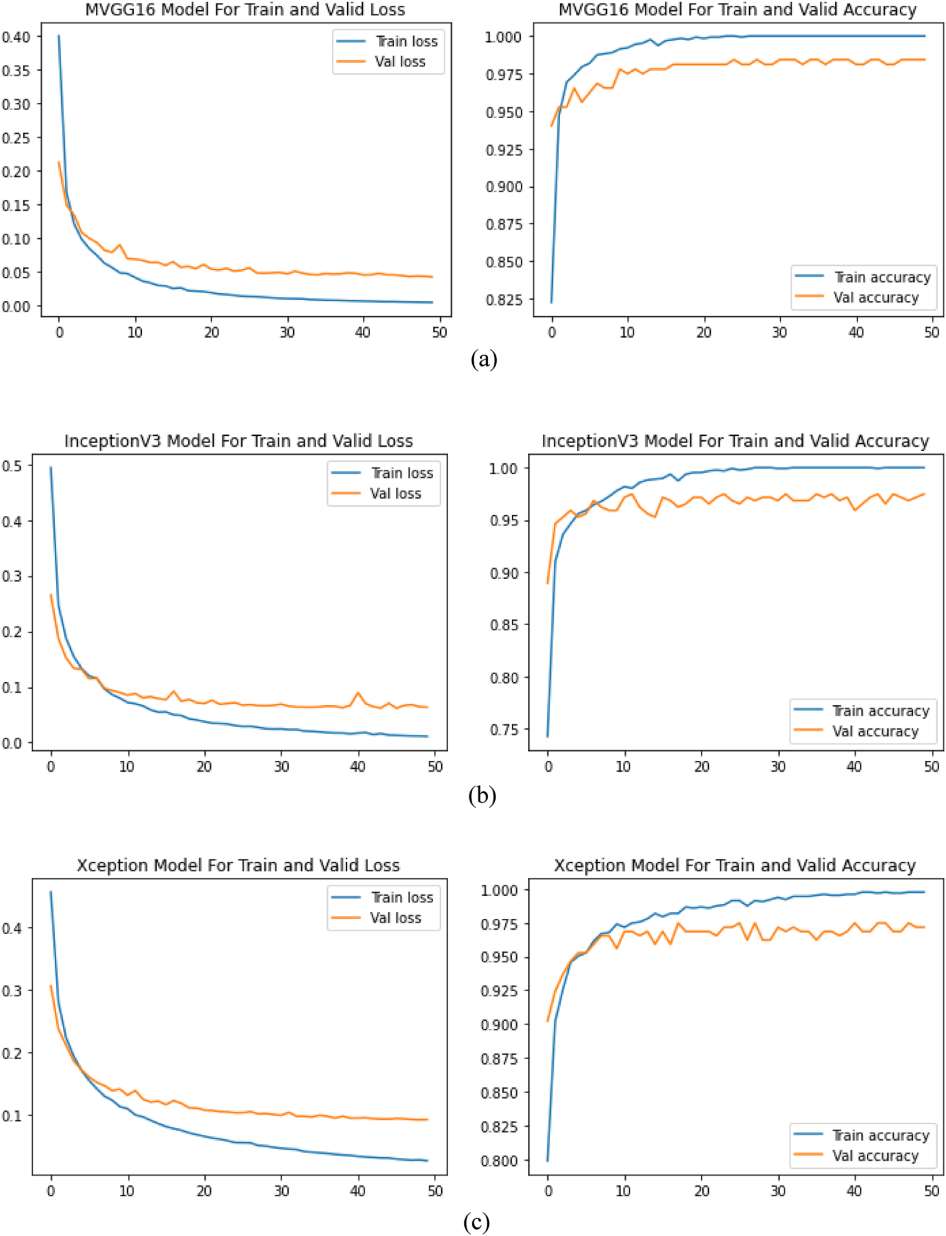
All the Model histories: (**a**) MVGG16; (**b**) InceptionV3; and (**c**) Xception.

Our model’s performance on the test set was 98.79% accuracy, 97.93% precision, 98.95% recall, 98.44% F1-scores, and 98.63% AUC, as opposed to 96.57% accuracy, 95.85% precision, 95.61% F1-score, 95.36% recall and 96.44% AUC for InceptionV3 and 96.57% accuracy, 94.82% precision, 95.56% F1-score, 96.32% recall and 96.25% AUC. Based on the testing set, a 98.79% accuracy, 97.93% precision, 98.95% recall, 98.44% F1-scores, and 98.63% AUC was obtained to evaluate the proposed model. The accuracy of the proposed MVGG16 was significantly higher than the two baseline models. These outcomes indicate the potency of our method for identifying habanero plant diseases. Three models, such as the proposed MVGG16, baseline InceptionV3 and Xception, were used for the implementation in this study. The training, validation and testing accuracies and losses of each model are presented in Table 1 and Fig. 2.

**Table 1:** Training, validation and testing accuracies and losses for the models.

Model	Training accuracy	Validation accuracy	Testing accuracy	Training loss	Validation loss	Testing loss
MVGG16	**1.000**	**0.9842**	**0.9879**	**0.0047**	**0.0424**	**0.0678**
InceptionV3	1.000	0.9748	0.9657	0.0110	0.0634	0.1110
Xception	0.9976	0.9716	0.9657	0.0272	0.0930	0.0817

Significant values are in [bold].

The system utilized dropout and regularization techniques to prevent overfitting. The model was trained at 50 epochs and used a batch size 32. The models were trained using Stochastic gradient descent (SGD) on the habanero plant dataset with a learning rate of 0.001 and a momentum of 0.9. The proposed MVGG16 CNN model outperformed the existing model with a training accuracy of 100%, validation accuracy of 98.42% and testing accuracy of 98.79%, as shown in Fig. 2 and Table 1. The training, validation and testing losses were 0.0047, 0.0424 and 0.0678, respectively, as seen in Table 1. This depicts that the proposed MVGG16 had the lowest losses over the other two models.

**Figure 3:**
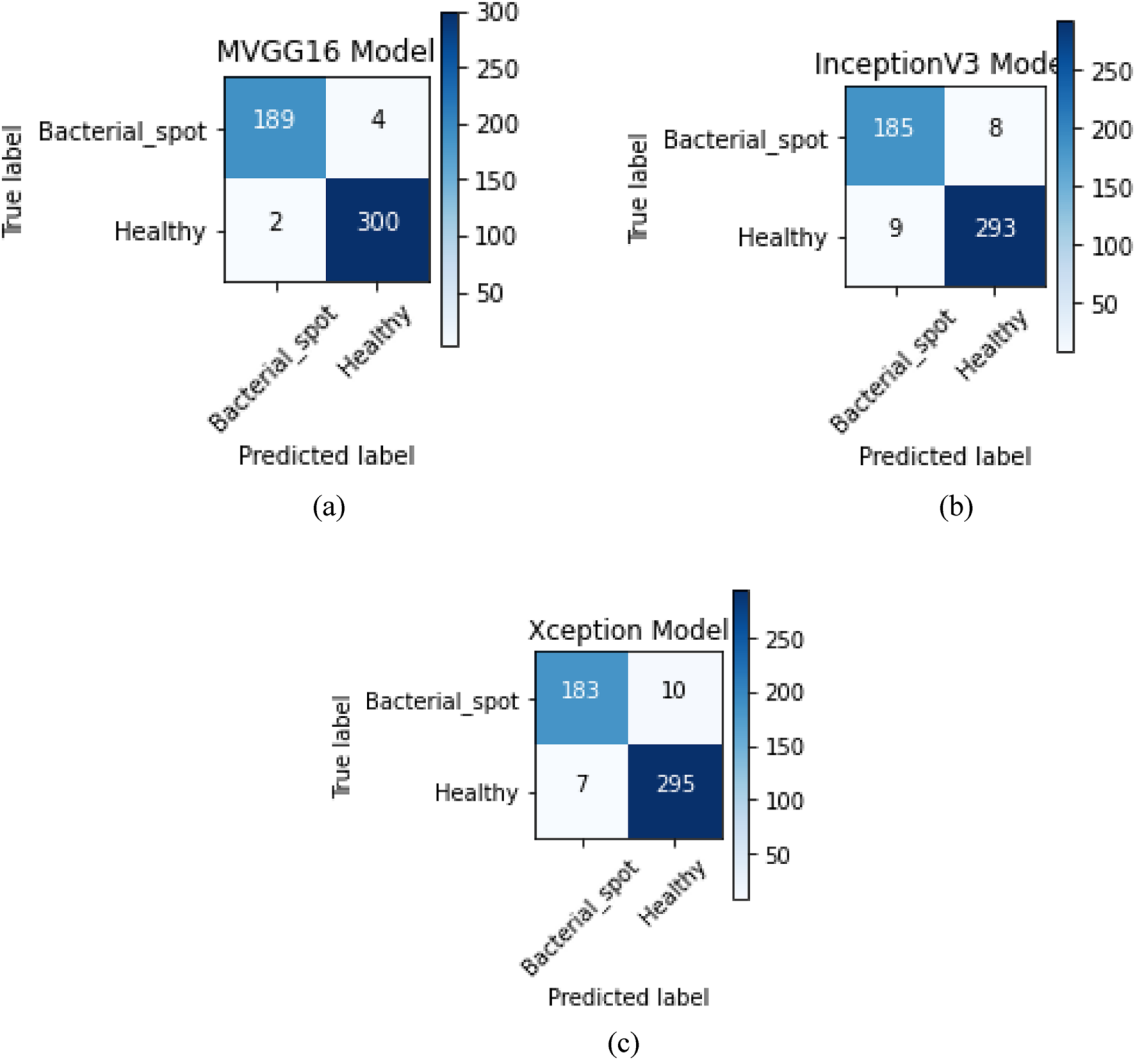
Confusion Matrix of the Models: (**a**) MVGG16; (**b**) InceptionV3; and (**c**) Xception.

Figure 3 shows the confusion matrix for the three models implemented, and it can be depicted in Table 2 that the proposed model had the best values for the TP, TN, FP, and FN. It was presented in both Fig. 3 and Table 2 that the proposed model had the highest TP and TN values of 189 and 300 with the lowest FP and FN values of 4 and, respectively.

**Table 2:** Confusion matrix table values for the models.

Model	TP	TN	FP	FN
VGG16	189	300	4	2
InceptionV3	185	293	8	9
Xception	183	295	10	7

**Figure 4:**
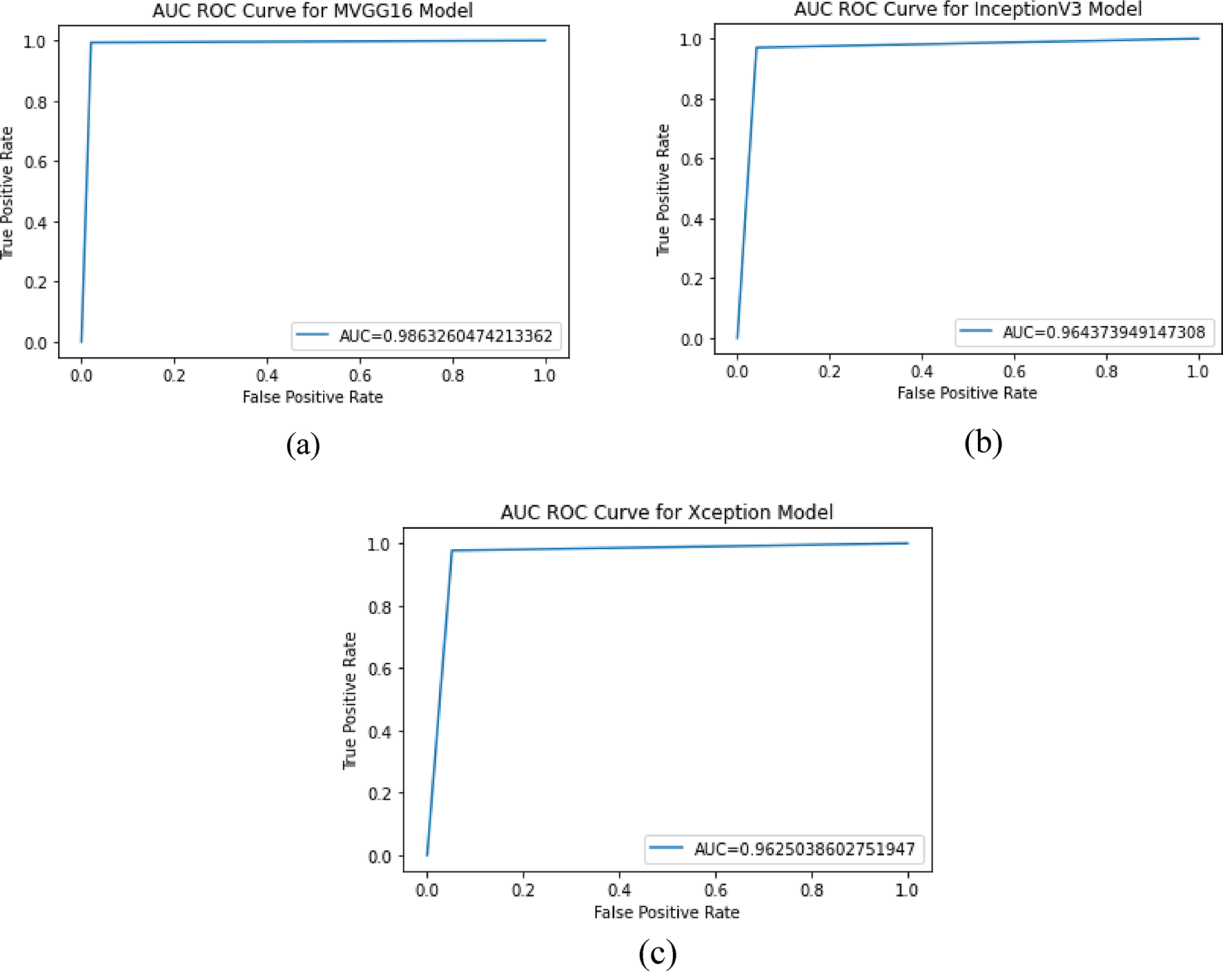
ROC-AUC curve for the models: (**a**) MVGG16; (**b**) InceptionV3; and (**c**) Xception.

The models were evaluated using five performance measures: accuracy, precision, f1-score, recall and AUC. It was presented in Table 3 that the proposed model outperformed the two models it was evaluated with a 98.79% accuracy, 97.93% precision, 98.44% f1-score, 98.95% recall and 98.63% AUC score. Figure 4 shows the ROC-AUC curve for the three models, and it is depicted that the proposed MVGG16 performed best.

**Table 3:** Performance evaluation of the MVGG 16 with baseline models.

Model	Accuracy	Precision	F1-score	Recall	AUC
MVGG16	**0.9879**	**0.9793**	**0.9844**	**0.9895**	**0.9863**
InceptionV3	0.9657	0.9585	0.9561	0.9536	0.9644
Xception	0.9657	0.9482	0.9556	0.9632	0.9625

Significant values are in [bold].

## Discussion

**Figure 5:**
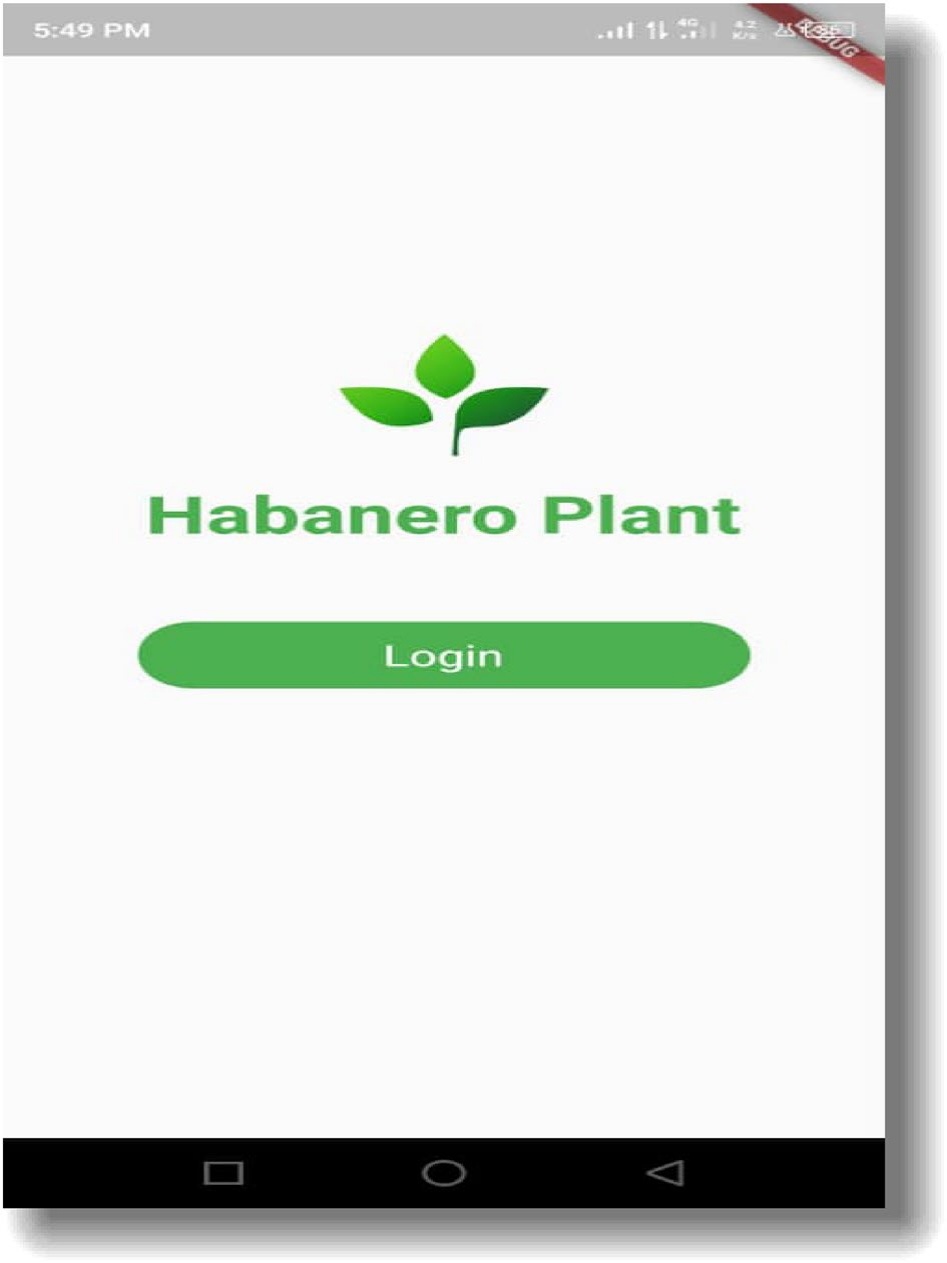
Smartphone Application Designed Interface.

**Figure 6:**
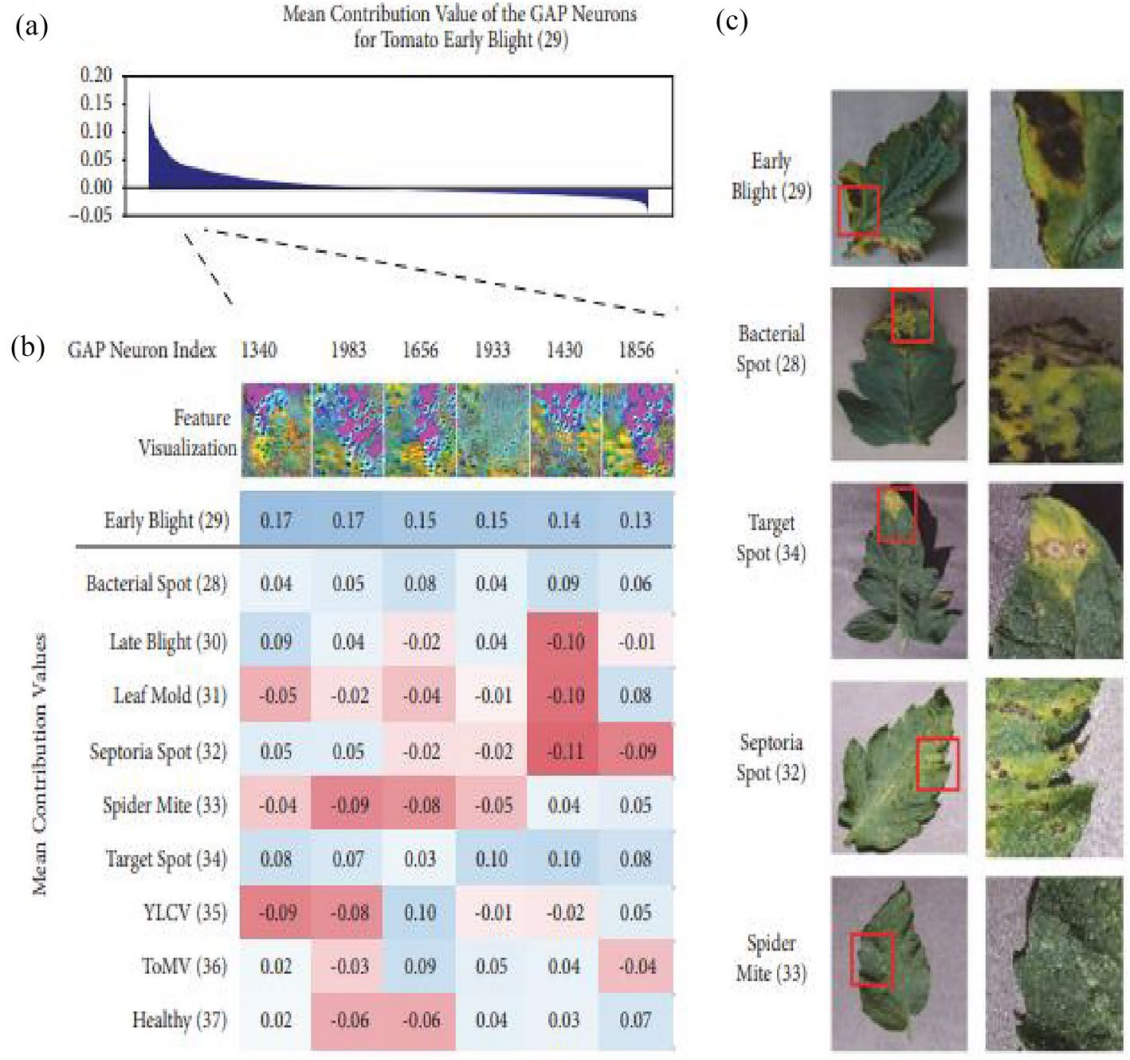
Plant Disease Visualization of Semantic Dictionary.

**Figure 7:**
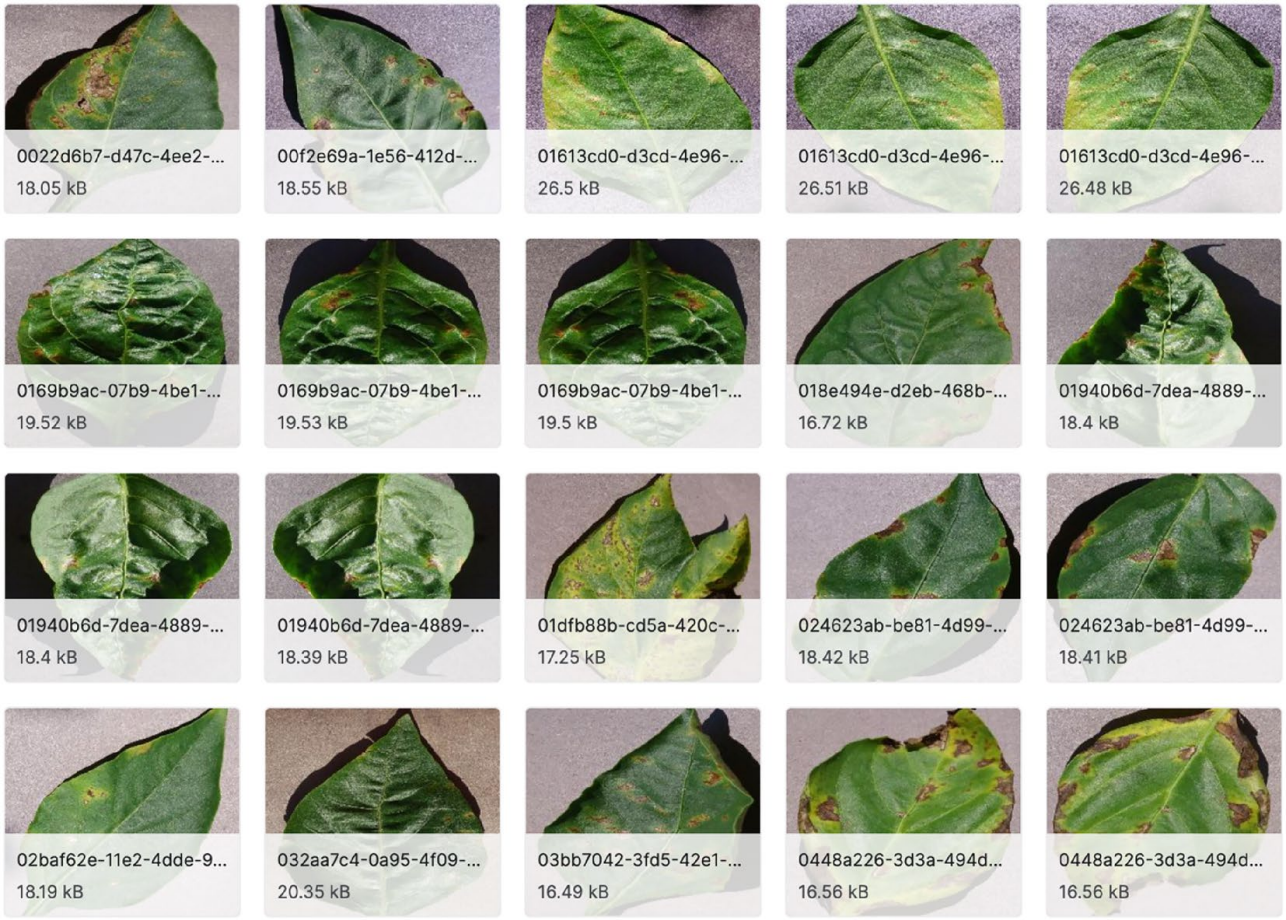
Testing Data Snapshot.

**Figure 8:**
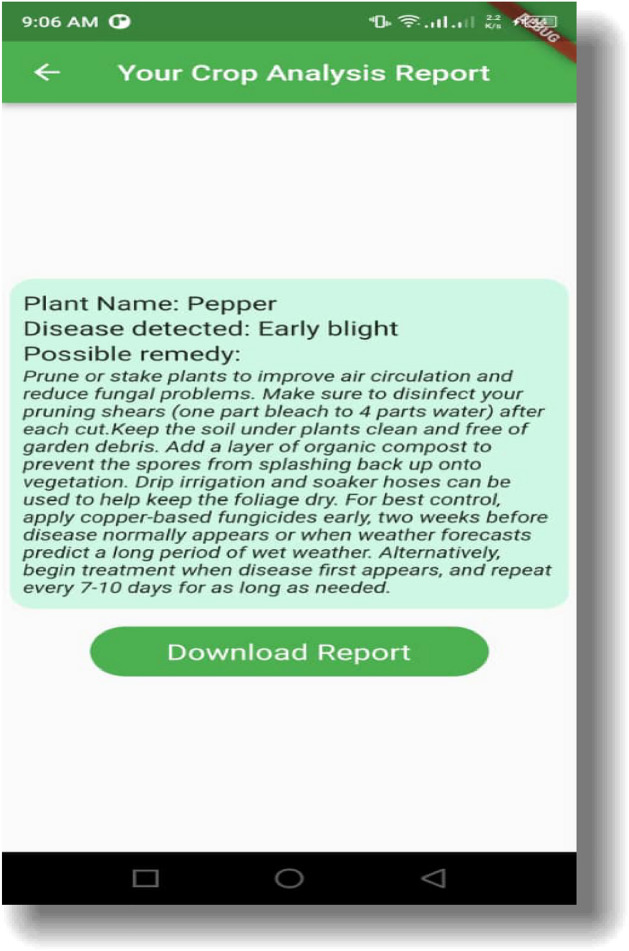
Report Generation from the User Input.

The article concludes with a discussion of the consequences of our research and future work, and Fig. 5 depicts the suggested application system interface. The habanero plant diseases dataset was acquired from the Kaggle repository, and image preprocessing and segmentation were conducted on the dataset. The diagram shown in Fig. 5 shows the proposed disease detection application system interface. This shows the home page of the mobile application where the user logs in to carry out the detection process. Figure 5 shows the login page interface of the smartphone design. The visualization of the semantic dictionary can be shown in Fig. 6. Figure 7 shows the snapshots for the testing dataset used in this study. Reports generated from the habanero disease inputted by the system users are shown in Fig. 8.

**Figure 9:**
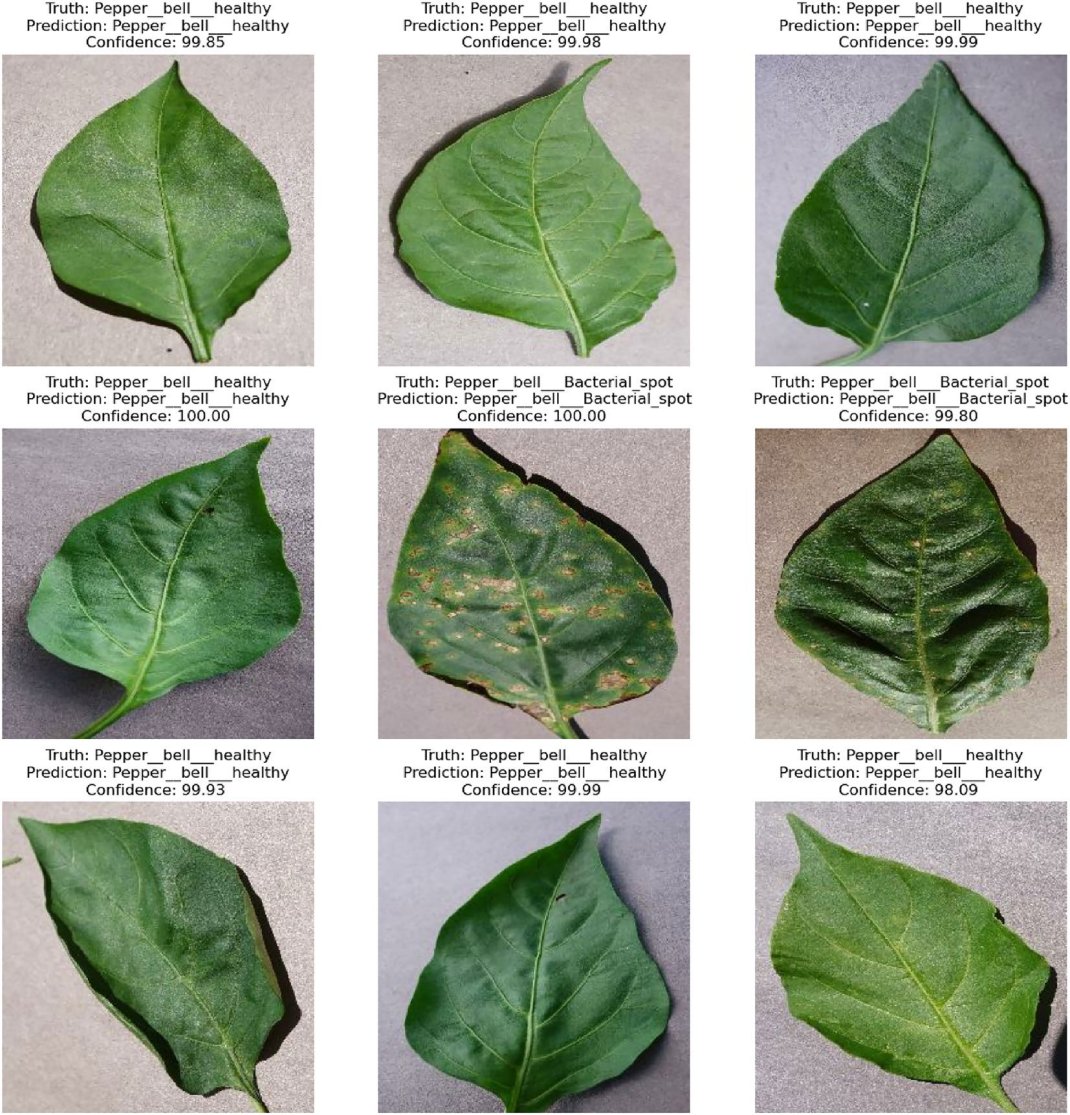
Prediction with a Confidence Rate of the Habanero Plant Diseases.

The confidence level was also used to evaluate the effectiveness of the proposed system, and it was discovered that the proposed MVGG16 CNN had a high confidence level from 99.00 to 100.00, as seen in Fig. 9. Twelve Habanero Plant Diseases datasets involving healthy and infected leaves were used to evaluate the system performance by prediction method. It can be seen in Fig. 9 that all the leaves selected for the prediction were predicted rightly when comparing them with the proper labels.

### Comparative analysis with related works

The proposed approach is similar to other studies that have used deep learning for plant disease detection. However, our study focused on habanero plants with unusual disease symptoms requiring specialized detection methods. Our results show that our model outperformed previous studies' accuracy results. Table 4 shows the comparative Analysis with existing studies that have examined the detection or classification of plant diseases. It can be demonstrated in the table that our proposed system outperformed all the existing methods with an accuracy of 98.42% over the studies conducted by Bezabih et al.^36^ with an accuracy of 95.82, one driven by Kumar, Razi, Singh & Das^37^ with an accuracy of 87.00% and third study conducted by Pant et al.^38^ with an accuracy of 96.00%.

**Table 4:** Comparative analysis with existing studies.

Authors	Year	Dataset	Models	Accuracy (%)
Bezabih et al.^36^	2023	Habanero leaf	VGG 16	95.82
Kumar et al.^37^	2020	Habanero leaf	mVGG 16	87.00
Pant et al.^38^	2021	capsicum plant (Bell pepper)	VGG	96.00
Proposed study	**2023**	Habanero leaf disease	**MVGG 16**	**98.42**

Significant values are in [bold].

### Limitations of the study

The limitation of the study is that it uses high computation resources such as space and time. The time complexity was too high because the modified algorithm layers increased, making the network deeper. Since the study is on mobile applications, it is suggested that in the future, mobile-based lightweight algorithms such as MobileNet, MobileNetV2, or V3 can be employed to detect plant disease.

## Conclusion

In this study, using an MVGG16 model, we created a smartphone app for detecting habanero plant disease. The software accurately distinguished between healthy and unhealthy plants, and in every instance where the plant was seriously infected, it could identify the precise ailment. These findings show how deep learning methods and smartphone technology can potentially increase the effectiveness and accessibility of plant disease diagnosis. To enhance the VGG16 model's performance on less prevalent diseases or plants in varying environmental circumstances, further research may concentrate on extending the dataset used to train the VGG16 model. Incorporating additional capabilities like real-time disease management advice or developing the software’s use for crops other than habanero peppers could also help the app get better.

As a result, we contend that the creation of this smartphone app for habanero plant disease detection is a significant advancement in the study of plant pathology and has the potential to be very helpful to farmers and agricultural experts in diagnosing and managing plant infections. The study can employ lightweight algorithms such as MobileNet versions, SqueezeNet, AlexNet, etc., to implement plant detection systems.

In the future, we intend to include two or more specific diseases, quick wilt caused by Phytophthora and slow wilt caused by Fusarium, and we want to expand the dataset to include these additional diseases to create a more comprehensive disease detection system.

## Data Availability

The data supporting this study's findings are available on Kaggle. The data can also be accessed at https://www.kaggle.com/datasets/arjuntejaswi/plant-village?resource=download.
